# Attentional modulation of contamination sensitivity: sharper perceptual boundaries for food compared to nonfood odor mixtures

**DOI:** 10.1093/chemse/bjag021

**Published:** 2026-07-28

**Authors:** Leonie Seidel, Astrid Endrup Iversen, Konstantina Kilteni, Janina Seubert

**Affiliations:** Department of Clinical Neuroscience, Karolinska Institutet, Stockholm, Sweden; Department of Psychology, Copenhagen University, Copenhagen, Denmark; Donders Institute for Brain, Cognition & Behaviour, Radboud University, Nijmegen, The Netherlands; Department of Neuroscience, Karolinska Institutet, Stockholm, Sweden; Department of Clinical Neuroscience, Karolinska Institutet, Stockholm, Sweden

**Keywords:** perceptual dominance, decision-making task, attention manipulation, deviation tolerance, choice curves, dilution series

## Abstract

Processing of contaminants in odor mixtures is influenced by their categorization into food and nonfood categories. For example, we previously found that participants were more likely to perceive food odors as contaminated by nonfood odors than vice versa, and that this effect was robust to changes in explicit task instructions. However, due to the mixing of food and nonfood components, processing differences between categories were not captured by this task design and thus remain to be explored. In the current pre-registered study, we address this knowledge gap by determining the effects of attention on the perception of odor mixtures containing only food or nonfood components. Participants (*N* = 24) assessed the perceived dominance of a target odor within an odor mixture. Response times to all odors increased toward the dilution series’ midpoint and were faster for food odor compared to nonfood odor mixtures. Perceived target dominance increased linearly for nonfood targets but exhibited a sigmoidal shape with a steeper slope (smaller just-noticeable difference) for food targets. This implies that distinguishing 2 food odors was easier than distinguishing between 2 nonfood odors, likely due to definitions higher relevance of distinct object category boundaries subserving contamination avoidance. At the same time, contamination sensitivity might be lower for nonfoods, allowing for greater tolerance of deviations. Attention did not influence the perception of nonfood but did so for ambivalent food mixtures and when the target was completely absent.

## 1. Introduction

Searching our surroundings for edible objects, we are faced with more information than we can consciously process. In general, our sensory systems deal with these large amounts of incoming information by jointly processing individual elements: configural perception is an information reduction method in which the brain integrates multiple pieces of incoming information to form a holistic percept or gestalt (Gottfried 2010; Coureaud et al. 2022). For instance, humans quickly and efficiently recognize faces by perceiving them as unified wholes, rather than encoding individual features like the eyes, nose, and mouth (Maurer et al. 2002; Taubert et al. 2011; Rossion 2013). Likewise, the processing of olfactory information is largely configural, as most environmental odors are not monomolecular but complex mixtures of many molecules (Gottfried 2010; Thomas-Danguin et al. 2014; Wilson et al. 2020). This processing mode explains why humans typically struggle to identify more than 4 individual components within an odor mixture (Laing and Francis 1989; Jinks and Laing 1999; Thomas-Danguin et al. 2014). However, this number varies depending on factors such as the individual’s level of training (Poupon et al. 2018), blending of odors (Livermore and Laing 1998), and the concentration ratios of the individual components (Kay et al. 2005; Wilson et al. 2020).

The tendency for configural processing enables humans to perceive odor objects as recognizable entities, which can be detected amidst a noisy odor background and under various contextual conditions (Gottfried 2010; Stevenson 2014; Thomas-Danguin et al. 2014; Coureaud et al. 2022). Recognition of objects is based on prior knowledge and permits their categorization into perceptual groups based on overlap in their associated cortical activation patterns (Howard et al. 2009; Gottfried 2010; Wilson and Sullivan 2011). While object-based perception is generally computationally efficient, there are certain situations in which focusing on individual components becomes essential. For example, an unfamiliar note appearing within a familiar odor, or an odor being encountered in an unexpected context can create a violation of expectation. In such cases, processing unexpected notes may be more crucial than accurately identifying an odor (Köster 2005; Møller et al. 2012), particularly when it comes to detecting food contamination or hazardous substances in the environment. Achieving such a shift requires a means of dynamically prioritizing certain sensory inputs over others, depending on the situation.

One flexible mechanism to deal with a noisy environment and ever-changing task demands is selective attention, which is the brain's ability to prioritize information most relevant to current goals. Much like tuning a radio to bring a faint broadcast into clarity while suppressing static, top-down attention allows us to amplify specific sensory inputs and dampen other, less relevant, signals. While this process is well-characterized in the visual and auditory systems, its role in olfaction remains less clearly defined (Veldhuizen 2017). Some studies have indicated a higher sensitivity to consciously attended odors, which is reflected in increased activation in piriform and insular cortex (Zelano et al. 2005, 2011; Plailly et al. 2008; Veldhuizen and Small 2011). Additionally, discrimination responses become more rapid when a cue has guided attention toward a stimulus (Spence et al. 2001). It has been suggested that attention, as a top-down modulator, can preactivate search templates in the piriform cortex, facilitating the detection of the attended odor within an odor mixture (Freeman 1981, 1983; Zelano et al. 2011).

As such, enhancing the perception of specific odors through regulation of top-down attention could offer behavioral advantages, such as helping to identify appetitive food cues or to detect subtle signs of spoilage. In a previous study, we investigated the effects of explicit attentional modulation on participants’ perception of food-to-nonfood odor mixtures as a theoretical framework for understanding the acceptance of inedible components (contaminants) in odors originating from a food object (Seidel et al. 2025). Participants showed a consistent bias toward categorizing mixtures as nonfood, an effect amplified under hunger, suggesting a potential protective mechanism of the olfactory system. Heightened contamination sensitivity under states of metabolic need may reflect stricter criteria for what is deemed edible, effectively narrowing the boundaries of the “food” category to improve the detection of potential threats. Strikingly, this nonfood bias persisted even when participants were cued to attend to the food component of a stimulus. This conflicted with earlier research demonstrating that participants were more likely to view odor mixtures as food-dominant when hungry, and that consuming food that matched the odor made these odor mixtures seem less food-like (Shanahan et al. 2021; Shanahan and Kahnt 2022). In contrast, our findings showed neither improved detection of an attended odor component nor increased food dominance in fasted states. One possible explanation is that the mixture of an edible food odor and a contaminant nonfood odor, particularly under fasted metabolic states, captured attention so effectively that it overrode the explicit task instruction. Alternatively, attentional top-down modulation may influence dominance perception differently for distinct odor categories.

Indeed, a substantial body of research indicates that food and nonfood odors differ in their perceptual, affective, and neural processing, pointing to higher ecological salience of food odors. Generally, detection of food odors seems to be faster than that of nonfood odors (Boesveldt et al. 2010; Seidel et al. 2025), and this is potentially driven by: (i) differential recruitment of liking and wanting components of reward (Savva et al. 2025), (ii) separate effects of repeated exposure on perceived pleasantness and intensity (Fontana et al. 2023), and (iii) distinct sensitivity to the effects of hunger and satiety (Albrecht et al. 2009; Shanahan et al. 2021; Nie et al. 2023). These category-specific differences underscore the functional relevance of accurately identifying food cues and raise the question of whether selective attention could modulate food and nonfood odor perception in distinct ways.

In the present study, we addressed this question by examining food and nonfood odor dilution series separately, thereby preventing categorical overlap and allowing a clearer test of whether top-down attention modulates perception of odor dominance differently depending on the category of the target stimulus. In line with previous research, we hypothesized that:

1. An odor would be more likely to be perceived as dominant as its relative concentration in a binary odor mixture increased (H1).
2. Decisions for pure odors would be faster than those for more ambiguous stimuli (H2).
3. Attending to an odor would enhance its perceptual dominance in a binary odor mixture (H3).
4. Attention might differentially modulate the perception of food and nonfood odors (H4; given that this hypothesis was exploratory, no direction was specified).

## 2. Methods

A pre-registration for this study can be found at the Open Science Framework (Seidel, L., Iversen A.E., & Seubert, J. (2024). Cognitive control over perceptual deviance processing: Separate effects of attention manipulation for food-to-food and non food-to-non food odor dilution series? https://doi.org/10.17605/OSF.IO/QM6KS). All procedures were in accordance with the Declaration of Helsinki and approved by the Swedish Ethical Review Authority (etikprövningsmyndighet, Dnr 2021-05138). A list of materials can be found in Supplementary Appendix A.

### 2.1. Participants

**Figure 1:**
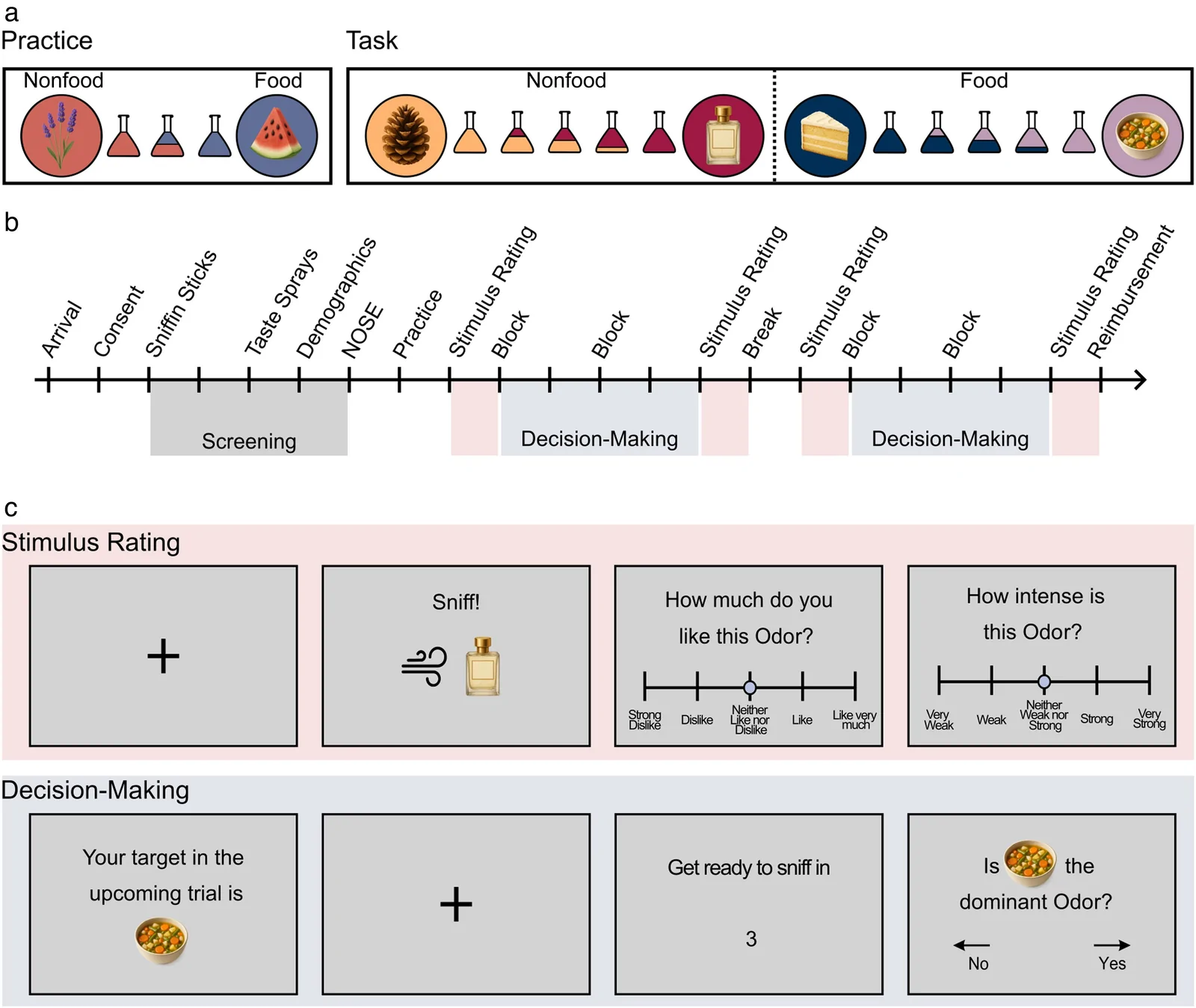
a) Participants received 3 odor dilution series, 1 for a practice run, 1 food, and 1 nonfood. b) After an initial screening, a short practice was run after which participants completed 4 blocks of an olfactory decision-making task using the odors displayed in (a). Two blocks were completed on food and nonfood odor dilutions, each time with one of its endpoints being presented as the target odor. Each vertical bar on the timeline represents ∼5 min; each block took ∼10 min. c) Stimulus ratings (pink on the timeline) were performed before and after blocks of the decision-making task (blue on the timeline). Fixation cross before the timer presented for a random duration between 3 and 7 s.

Healthy participants (Table 1), with normal or corrected-to-normal vision, participated in the study. The sample size (*N* = 24) was determined as part of a larger pre-registered project and was not based on an a priori power analysis. Participants were recruited until a final sample of 24 individuals meeting all inclusion criteria was obtained. Exclusion criteria included smoking/snus use, diagnosis of a psychiatric disorder, or body mass index (BMI) <18.5 or >24.9. Recruitment was done at universities in Stockholm and through the Sona recruitment system. Participants were instructed to abstain from eating and drinking anything except water for at least 1 h before the study session. Olfactory dysfunction was assessed through the 16-odor identification sub-test of the Sniffin’ Sticks (Hummel et al. 1997) test (exclusion: >5 mistakes) and the nasal obstruction symptom evaluation (Stewart et al. 2004) questionnaire (exclusion: score ≥55). Potential taste dysfunction was measured through the identification ability of taste sprays (Vennemann et al. 2008) for the basic tastes sweet, salty, sour, and bitter (exclusion: ≥1 mistake). In total, 29 individuals were screened for participation (Fig. 1b); 5 did not meet the inclusion criteria (3 based on odor identification scores and 2 based on BMI) and were therefore not included in the study. All participants provided written informed consent and received gift vouchers valued at 200 SEK upon study completion.

**Table 1:** Sample descriptives.

	All (*N* = 24)			Women^a^ (*n* = 15)			Men^a^ (*n* = 9)		
	M	SD	Range	M	SD	Range	M	SD	Range
Age^b^	25.292	3.507	19 to 35	24.733	2.789	21 to 31	26.222	4.494	19 to 35
Education^b^	16.375	1.974	12 to 19	16.200	1.859	12 to 19	16.667	2.236	12 to 19
BMI	22.613	1.868	18.8 to 24.9	22.127	2.071	18.8 to 24.9	23.422	1.162	21.7 to 24.8
Hunger^c^	3.167	0.702	2 to 4	3.000	0.655	2 to 4	3.444	0.726	2 to 4
Alertness	74.208	13.828	50 to 99	72.533	15.528	50 to 99	77.000	10.665	60 to 94
NOSE	31.042	7.369	25 to 50	30.000	6.268	25 to 45	32.778	9.052	25 to 50

^a^Self-reported gender among female/male/other (open ended).

^b^In years.

^c^On rating scale 0 to 100.

NOSE, nasal obstruction symptom evaluation.

### 2.2. Stimulus material and stimulus presentation

Three food and 3 nonfood odors were used in this study (Fig. 1a). The food odors were watermelon (57.14% v/v in propylene glycol), cake (40% v/v in 5% v/v ethanol), and vegetable broth (1% w/v in 4.35% v/v ethanol). The nonfood odors were lavender (57.14% v/v in propylene glycol), pine (12% v/v in propylene glycol), and floral-earthy perfume (33.33% v/v in 32% v/v ethanol). Odor concentrations were adapted from a previous study (Seidel et al. 2025) and piloted to be of equal perceived intensity. Stimuli were prepared freshly every week and kept in a fridge at 5° in between testing sessions. From these 6 stimuli, 3 odor dilution series were created. Lavender, watermelon and a 50%/50% midpoint between the two were used as a training set to practice the instructions of the perceptual decision-making task. The remaining 2 dilution series were of cake with vegetable broth, and pine with perfume, with 5 steps each (0%/100%, 25%/75%, 50%/50%, 75%/25%, 100%/0%), which were used during the main task. During each block of the experiment, 1 pure odor was presented as the target, whose perceptual dominance was to be evaluated on each trial. The odor stimuli were presented using a computer-controlled 16-channel olfactometer (Whiff Inc., adapted from Lundström et al. [2010]). The airflow was directed via tubing attached to computer-controlled solenoid valves through the odorized headspace of odor jars (at 3 L/s) containing 3 mL of the odorant. Odorized air was then presented orthonasally to the participants, with nose pieces inserted into the nostrils. Two of the 16 channels were used for nonodorized air: 1 channel was opened in between odor presentations. Another channel produced a constant low-pressure airflow (at 1 L/s) masking the audible clicking and tactile “puff” sensed from the nosepiece when air and odor channels were switched (Boesveldt et al. 2010; Miller et al. 2013; Seubert et al. 2015).

### 2.3. Physiological data: respiration

The ADInstruments PowerLab C and the corresponding C Series instrument interface were used to record participants’ respiration. A nasal temperature probe was attached to 1 nosepiece and placed inside the participant's nostril to record the decreasing temperature of inhalations and increasing temperature of exhalations. Temperature probe output was amplified and filtered.

### 2.4. Perceptual decision-making

Participants completed a computerized olfactory perceptual decision-making task (Fig. 1c) implemented in PsychoPy (Peirce et al. 2019). After a verbal explanation of the task instruction, participants completed a short practice run using the training odor set. The main task consisted of 4 blocks (35 trials each, 7 repetitions per stimulus), 2 on the food (cake to vegetable broth), and 2 on the nonfood (pine to perfume) dilution series. Whether a participant first completed the food or nonfood blocks was randomized, as was the block order within each dilution series. At the beginning of the experiment, odors were familiarized in the context of a stimulus rating procedure. During this olfactory stimulation, visual stimuli that were congruent with the odors (Blechert et al. 2019; Toet et al. 2019) were presented on the screen. This helped participants to understand what odor they were smelling without relying on verbal labels. Participants were then instructed to rate the odors’ intensity (“How intense is this odor?”) and pleasantness (“How much do you like this odor?”) on visual analog scales ranging from 1 (“very weak” or “strong dislike”) to 5 (“very strong” or “like very much”). Importantly, this familiarization occurred in a block-relevant manner: odors associated with the first pair of blocks were rated before block 1 and again after block 2, whereas odors associated with the second pair of blocks were rated before block 3 and again after block 4.

At the beginning of each block, 1 of the 2 odors was presented as the target by showing the previously associated picture on the screen. Participants then had to determine the target's perceptual dominance on each trial using a forced-choice format with the options “yes” (dominant) or “no” (not dominant). On each trial, a random odor from the dilution series was presented until the participants had made their decision. If no decision was made within 40 s, the task advanced and the trial was discarded. At the end of the second block, participants were instructed to rate the 2 target odors again for pleasantness and intensity. After a 5-min break, participants completed the remaining 2 blocks in the same way, smelling food odors if the previous 2 blocks had presented nonfood odors and vice versa (Fig. 1b).

## 3. Analysis

### 3.1. Stimulus ratings

We aimed to demonstrate that (i) at baseline, the 4 odors used in the study did not differ meaningfully in pleasantness or intensity and (ii) ratings within an odor did not differ pre- to post-task, as a result of prolonged odor exposure. We employed equivalence testing using the *TOSTER* package (Lakens 2017; Lakens et al. 2018) to formally test whether any differences in ratings were small enough to be considered negligible. Non-normality of data was addressed by employing bootstrapped equivalence testing. Equivalence bounds were set at Cohen's *d* = ± 0.597, representing the smallest effect size we would be able to detect with 80% power, and effects falling within the constructed region were considered statistically equivalent.

### 3.2. Perceptual decision-making

For quality control, responses were visually inspected, separately for each block, and averaged over target identity (food vs. nonfood). With increases in target concentration, increases in perceived target dominance were expected. Two participants were excluded because their response patterns indicated reversed behavior in some blocks, suggesting they may have failed to understand the target odor reversal. We then fitted 2 separate mixed-effects regressions to responses and their associated response times (RT) using the *lme4* package (Bates et al. 2015). The *response model* was specified with a binomial (logit) link function (target odor dominant “yes” vs. “no”) and a BOBYQA optimizer. To the *RT model* (nplotwrap optimizer) we added both a linear and a quadratic term of target concentration to capture the data's inverted U-shape. A positive skew in the RT data was addressed by log-transforming the outcome variable. In both models, perfect collinearity (aliasing) occurred for block (broth, cake, pine, perfume), and target (food, nonfood), where the former was nested within the latter, and this was addressed by letting target be a fixed effect and estimating the variation around it by including a random block slope. To address an ongoing debate about different olfactory abilities between men and women (Sorokowski et al. 2019), we added gender to the models to correct for any such effects. The resulting models were simplified through backwards deletion, comparing model fits (Meteyard and Davies 2020) after each reduction step (Supplementary Appendices B and C). While our model selection was guided by the data, in the RT model, we retained 1 theoretically motivated interaction (target concentration × target) that approached significance (*P* = 0.055). Given the visual trends in the data and the only marginal improvement (Δχ^2^(1) = 3.69, *P* = 0.055) in model fit when removing the term, we chose to include the interaction to avoid discarding a potentially meaningful effect. For the final models of both variables, optimizer checks were performed using the *allFit()* function (Supplementary Appendix D), to ensure that our models’ parameters were not dependent on the chosen optimizers.

### 3.3. Parameters from the psychometric function

**Figure 2:**
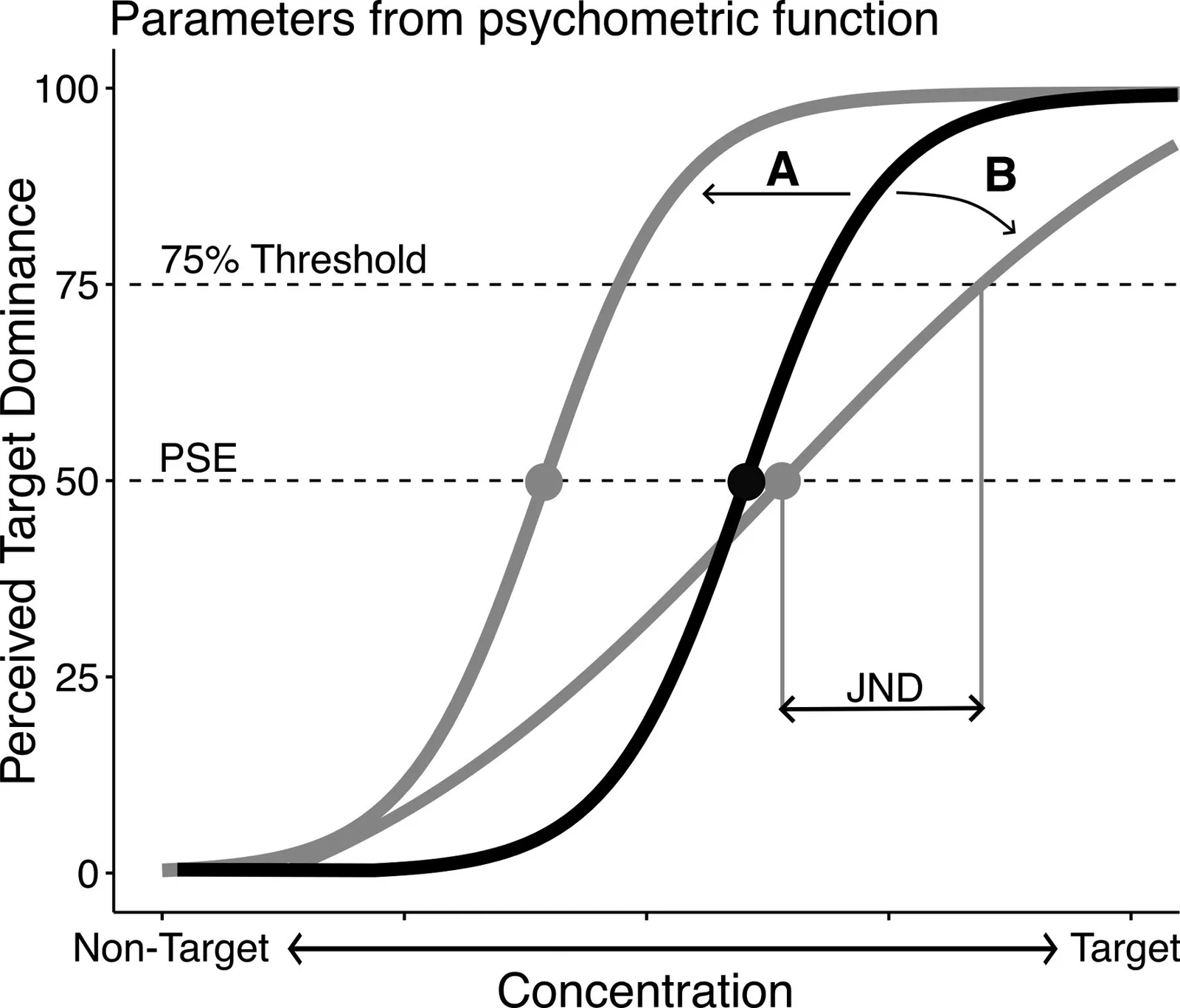
Conceptual illustration of the psychometric function and key parameters. Perceived target dominance (*y*) increases as a function of target concentration (*x*) (H1). a) A leftward shift of the function reflects a decrease in the point of subjective equality (PSE), indicating that the attended odor is perceived as dominant at lower concentrations (H3; exploratory H4). b) A flatter slope corresponds to an increased just noticeable difference (JND; i.e. a larger difference between 50% and 75% “yes” responses), reflecting greater task difficulty and/or reduced perceptual separability between the odors (exploratory H4).

Next, we fitted separate sigmoid psychometric (i.e. physical stimulus—response) functions (equation 1) to individual response data in each of the 4 target blocks, allowing us to investigate 2 parameters of interest (Fig. 2).


(1) $$P(\mathrm{response})=\frac{1}{1+e^{-\left(\beta 0\;+\;\;\beta 1x\right)}}$$

To assess monotonicity, we examined the proportion of “yes” responses at the 2 endpoints of each dilution series. We considered responses plausible when they fell into the expected quadrants of the function. Specifically, 0 ≤ *P*(yes) ≤ 0.5 for 0% target concentration, and 0.5 ≤ *P*(yes) ≤ 1 for 100%. Three participants were excluded because their response behavior was flat/nonmonotonic, so that any parameters extracted from fitted response functions would be uninterpretable and/or fall outside the tested stimulus range. For the remaining participants (*n* = 19), we extracted the model's beta coefficients (β_0_, β_1_) and computed individual points of subjective equality (PSE) and just-noticeable difference (JND) values. The PSE (equation 2) is associated with the location of the curve and thus indicates response bias through shifts along the *x* axis (Knoblauch and Maloney 2012; McCarley and Yamani 2021). It corresponds to the concentration at which the target odor is perceived as dominant on 50% of trials. A lower PSE implies that the target is perceived as dominant even at low concentrations.


(2) $$\mathrm{PSE}=-\frac{\beta 0}{\beta 1}$$

The JND (equation 3) is used as a measure of stimulus discriminability and is related to the slope of the curve. More specifically, this parameter indicates the smallest difference between 2 stimuli that the participant is able to detect, and a steeper slope is therefore reflective of better stimulus separation (Gescheider 1997; Kingdom and Prins 2016; McCarley and Yamani 2021).


(3) $$\mathrm{JND}=\frac{\log(3)}{\beta 1}$$

Distributions of PSE and JND did not meet the assumption of normality and were thus analyzed through Holm-corrected Wilcoxon sign-rank tests.

### 3.4. Exploratory analysis of attention

Both previous analysis steps examined the qualitative differences in how food and nonfood odor mixtures are perceptually processed. To investigate how attentional focus may differentially influence perceptual dominance for these categories, we compared the proportion of perceived target dominance across the 2 blocks in which the same physical odor was presented under different target instructions. For instance, we assessed how often participants identified the target as dominant for a stimulus composed of 25% cake and 75% broth when focusing on cake versus when focusing on broth. Importantly, we chose to analyze only “yes” responses because a “no” response does not logically imply that the nontarget odor was dominant. For each participant and stimulus mixture, the proportions of “yes” responses was summed over the 2 target blocks and analyzed through Wilcoxon signed-rank tests. This allowed us to test these rival hypotheses:

1. If perceptual dominance is purely stimulus-driven, the summed proportions should reflect physical target concentrations and approach 1.
2. If attending to an odor perceptually amplifies it, the proportion of “yes” should be higher than the target's physical concentration, so that a sum over the 2 blocks should be >1.
3. If attending to an odor perceptually attenuates it, the proportion of “yes” should be lower than the target's physical concentration, so that a sum over the 2 blocks should be <1.

### 3.5. Physiological data: respiration through nasal temperature

**Figure 3:**
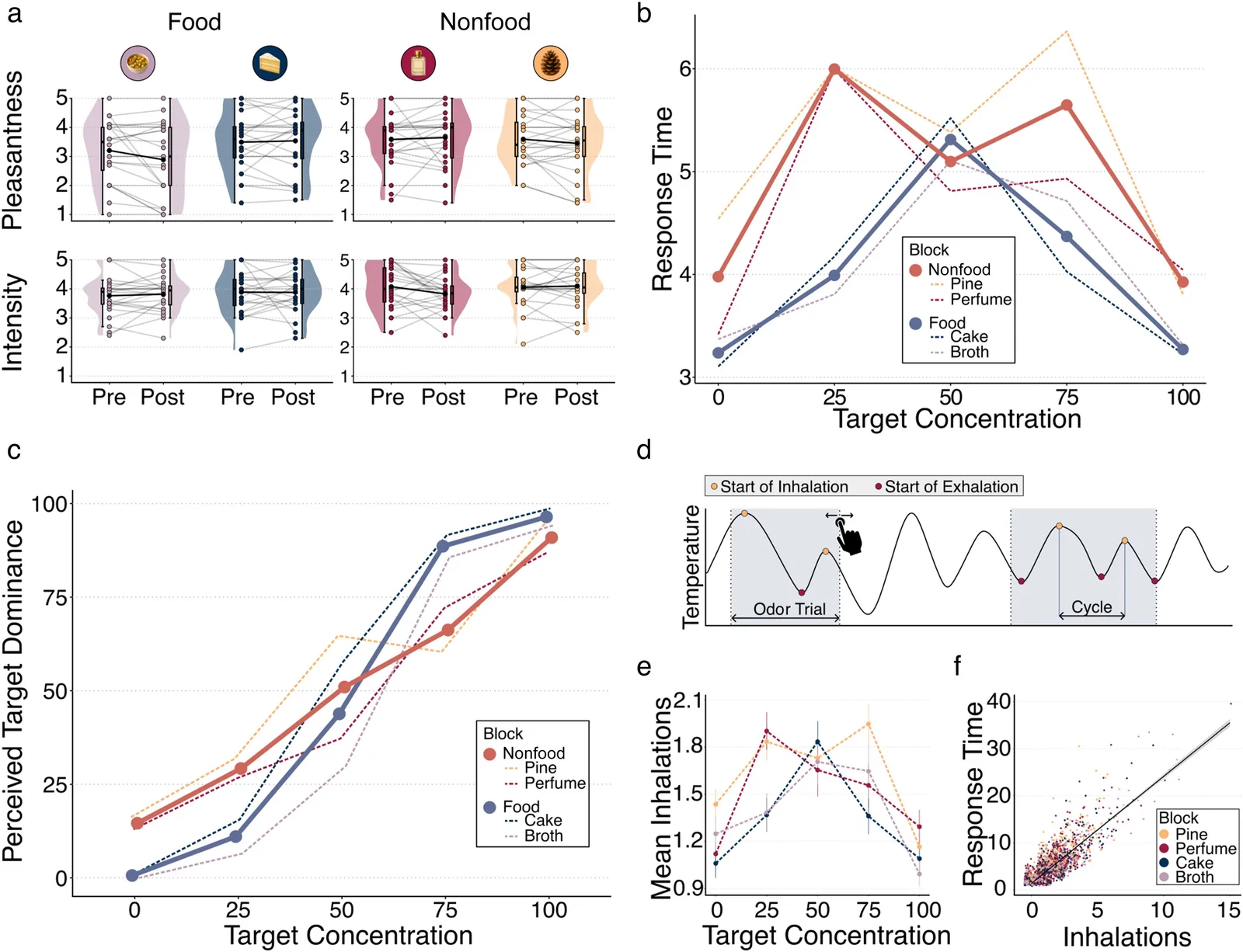
a) Ratings of odor pleasantness and intensity did not show significant changes from pre- to post-task, indicating no strong influences of repeated exposure. b) Response times averaged over the blocks within a target category (bold), indicated faster decision-making for food than nonfood. Decisions were faster for pure target odors, and slowest for around the dilution series’ midpoints. c) Observed responses over the target concentration spectrum verify a basic increase of target dominance at higher concentrations. d) A trial was defined by the odor onset and the time-point of a participant's button press (response). During the odor presentation window, the number of inhalations was tracked based on peak nasal temperature recorded from the nostril. e) The mean number of inhalations follows the same pattern as the recorded response time (b), indicating that RT is a valuable proxy for evidence sampling in an odor decision-making task. f) Number of inhalations and RT are strongly correlated.

Nasal temperature was acquired in Labchart as a physiological measure of respiration, where increases and decreases in temperature reflect expiration and inspiration, respectively (Schaefer et al. 2024). The temperature signal was recorded at a frequency of 200 Hz and underwent cleaning and pre-processing using the *compute_respiration()* function from the Python *Physio Toolbox* (Ghibaudo et al. 2023). The function was tailored to the signal by applying a band-pass filter in the range of 0.03 to 1 Hz, while all other parameters were maintained at their default settings. Defined by stimulus onset and offset markers, we detected respiratory cycles and extracted the number of inspirations, identifying cycles based on the troughs and peaks (extrema) of the filtered signal (Fig. 3d). Extracted breathing parameters were subsequently visualized and correlated with behavioral outcomes.

## 4. Results

### 4.1. Stimulus ratings

*Pleasantness* ratings (Table 2) were largely stable across timepoints and odors (Fig. 3a). From *pre-task to post-task,* cake, perfume, and pine showed statistically equivalent ratings (all *P* < 0.05). In contrast, the perceived pleasantness of broth was higher before the task, resulting in nonequivalent timepoints (*t*_equiv_(23) = −0.76, *P* = 0.23). At the *first timepoint* of assessment, ratings of several odor pairs showed statistically equivalent pleasantness (all *P* < 0.05). Only the comparisons of broth vs. pine (t_equiv_(23) = 1.14, *P* = 0.12, t_null_(23) = −1.79, *P* = 0.06) and broth vs. perfume (t_equiv_(23) = 1.46, *P* = 0.08, t_null_(23) = −1.46, *P* = 0.14) were neither statistically equivalent nor statistically different. Taken together, we found no strong evidence of systematic differences in perceived pleasantness across odors or timepoints.

**Table 2:** Stimulus ratings at both timepoints.

Pleasantness				Intensity			
Pre-task		Post-task		Pre-task		Post-task	
Mdn	IQR	Mdn	IQR	Mdn	IQR	Mdn	IQR
3.500	1.470	3.000	2.000	3.900	0.550	3.950	0.650
3.600	1.080	3.900	1.250	4.000	0.900	4.000	0.825
3.900	0.925	4.000	1.200	4.000	1.180	3.850	0.625
3.400	1.170	3.550	1.030	4.050	0.500	4.050	0.700

Likewise, *intensity* ratings (Table 2) indicated no substantial differences between the odors (Fig. 3a). From *pre- to post-task*, ratings were equivalent for broth, cake, and pine (all *P* < 0.05), indicating that repeated exposure did not change perceived odor intensity. For the perfume odor, results were inconclusive (*t*_equiv_(23) = −1.22, *P* = 0.10, *t*_null_(23) = 1.71, *P* = 0.91). Comparisons between odors at the *pre-task timepoint* revealed statistically equivalent intensity ratings for several pairs (*P* < 0.05), with the only exception being broth vs. pine (*t*_equiv_(23) = 1.36, *P* = 0.07, *t*_null_(23) = −1.57, *P* = 0.14) and broth vs. perfume (*t*_equiv_(23) = 1.30, *P* = 0.09, *t*_null_(23) = −1.63, *P* = 0.09), where results were inconclusive. Overall, we found no clear evidence that the 4 target odors differed in intensity prior to the task, nor that their perceived intensity changed meaningfully over time. A full summary of all equivalence and corresponding null hypothesis tests can be found in Supplementary Appendix E.

### 4.2. Perceptual decision-making

The final model of log-transformed *response time* (Table 3) explained approximately 30% (conditional R^2^) of variance in the data. Large random effects contributions reflected (i) between-subject differences in RT and (ii) varying effects of block across participants (Supplementary Appendix F). The model's intercept was estimated at 1.038 on the log scale, corresponding to a back-transformed RT of 2.83 s for the reference category (food target absent, female participant responds nondominant). The inverted-U shape pattern for RT (Fig. 3b), where responses are slower for mixtures than pure odors (H2), was captured by both a linear and a quadratic effect of target concentration: RT first increased with increasing target concentration (linear effect) and then decreased again at higher concentrations (quadratic effect). This pattern likely reflects perceptual ambiguity near the midpoint of the dilution series. A marginal interaction with target indicated that this concentration effect might be different for food and nonfood target odors. At the reference category (i.e. no target present), RT was ∼30% slower for nonfood (e^0.254^) compared to food targets. The effect of concentration was also slightly attenuated (e^−0.01^ = 0.99) for nonfoods. Overall, this suggests that RT increased with ambiguity for both target types, but more steeply so for food targets.

**Table 3:** Mixed effects regression model of log-transformed response time.

	Response time					
Predictors	b*	SE	95% CI	β*	Statistic	*P*
(Intercept)	1.038	0.093	(0.856, 1.219)	−0.050	11.213	**<0**.**001**
Target concentration	0.137	0.010	(0.117, 0.157)	0.739	13.419	**<0**.**001**
Target (nonfood)	0.254	0.062	(0.134, 0.375)	0.309	4.128	**<0**.**001**
Gender (man)	0.053	0.129	(−0.201, 0.306)	0.080	0.408	0.684
(Target concentration)^2^	−0.013	0.001	(−0.015, −0.011)	−0.730	−14.209	**<0**.**001**
Response behavior (yes)	−0.024	0.026	(−0.076, 0.028)	−0.036	−0.900	0.368
Target concentration × Target (nonfood)	−0.010	0.005	(−0.021, 0.000)	−0.056	−1.918	0.055
**Random effects**						
	Variance		SD			
σ^2^	0.279					
τ_00 participants_	0.090		0.300			
τ_11 participants blockPerfume_	0.061		0.247			
τ_11 participants.blockCake_	0.076		0.275			
τ_11 participants.blockBroth_	0.085		0.291			
ICC	0.240					
*N*_subj_idx_	22					
Observations	3,077**					
Marginal R^2^/Conditional R^2^	0.074/0.299					

Bold values refer to significance at *P* < 0.05.

^*^Note: b is the unstandardized coefficient, and β is the standardized coefficient.

^**^Three trials discarded because participants failed to respond within the decision window.

The final *response* model (Table 4) explained a substantial portion of the variance in responses given on each trial (conditional R^2^ = 0.747), with the fixed effects alone accounting for most of it (marginal R^2^ = 0.579). Generally, perceived target dominance increased with increasing target concentration (Fig. 3c). At the reference level (food target odor, concentration 0%, female participant, average RT), the chance of perceiving the target odor as dominant was very low, about 0.5% $\left(P(\mathrm{yes})=\frac{e^{-5.279}}{1+e^{-5.279}}\;\right)$. Crucially, and in line with our research question, the model revealed a significant interaction between target type and target concentration, alongside significant simple effects of both predictors: When the target concentration was 0%, participants were approximately 20 times less likely to identify a food target odor as dominant than a nonfood target. As concentration increased, food odor targets became increasingly likely to be perceived as dominant. Thereby, each 10% increase in target concentration (i.e. 1-unit increase in the scaled concentration predictor) was associated with a ∼2.7-fold increase in the odds of a “yes” response (H1). For nonfood targets, this concentration effect was substantially smaller, with an odds ratio of approximately 1.6 (e^0.990 to 0.524^). These findings highlight differential deviance processing for food and nonfood odors (H4).

**Table 4:** Mixed effects regression model of response behavior.

	Response behavior					
Predictors	Log-odds	SE	95% CI	OR	Statistic	*P*
(Intercept)	−5.279	0.435	(−6.13, −4.426)	0.005	−12.132	**<0**.**001**
Target concentration	0.990	0.079	(0.835, 1.145)	2.691	12.512	**<0**.**001**
Target (nonfood)	3.030	0.364	(2.315, 3.744)	20.694	8.312	**<0**.**001**
Gender (man)	−0.070	0.165	(−0.393, 0.253)	0.932	−0.425	0.671
Response time	−0.069	0.051	(−0.169, 0.031)	0.933	−1.358	0.174
Target concentration × Target (nonfood)	−0.524	0.053	(−0.628, −0.420)	0.592	−9.894	**<0**.**001**
**Random effects**						
	Variance		SD			
Residual σ^2^	3.290*					
τ_00 participants_	2.195		1.481			
τ_11 participants blockPerfume_	4.811		2.193			
τ_11 participants.blockCake_	2.646		1.627			
τ_11 participants.blockBroth_	3.356		1.832			
ICC	0.400					
*N*_participants_	22					
Observations	3,077**					
Marginal R^2^/Conditional R^2^	0.579/0.747					

Bold values refer to significance at *P* < 0.05.

^*^Note: For logistic mixed models, σ^2^ is not estimated from the data but is fixed at π^2^/3 ≈ 3.29, corresponding to the variance of the standard logistic distribution.

^**^Three trials discarded because participants failed to respond within the decision window.

### 4.3. Parameters from the psychometric function

**Figure 4:**
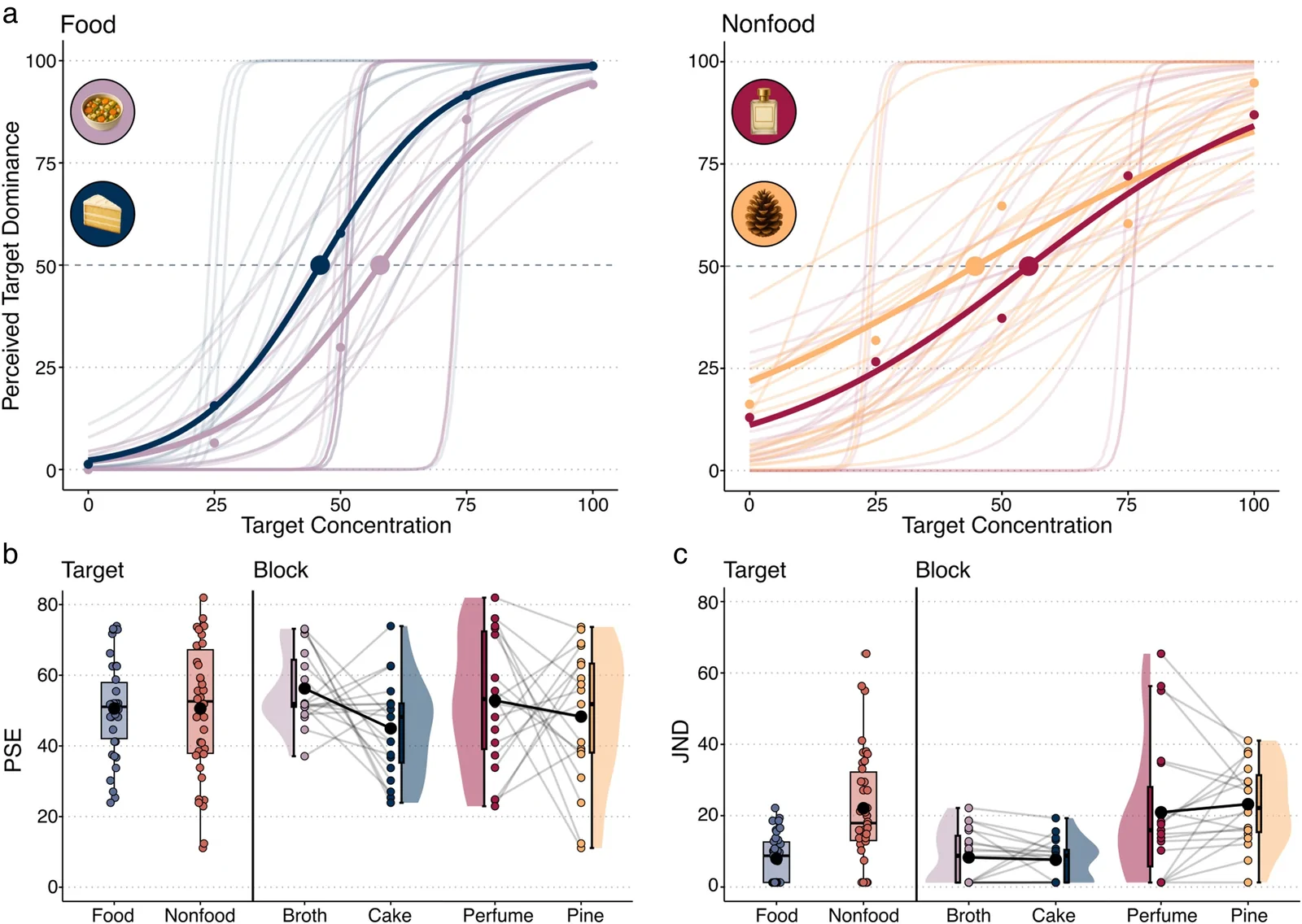
a) Psychometric stimulus-response functions were fitted to individual data for both the food and nonfood odor dilution series. Group data are indicated in bold. Steeper slopes indicate better perceptual separation of the 2 foods, compared to the 2 nonfood odors. b) PSEs, as indicated on the 50% line in (a), did not differ between the blocks. c) JNDs were higher in nonfood compared to food but did not differ between blocks of the same target category.

McFadden's pseudo R^2^ values (McFadden 1977), which averaged 0.548 (SD = 0.244) across all conditions, indicated moderate to good fit overall. However, model fit varied substantially by block (M ± SD: broth = 0.674 ± 0.192; cake = 0.693 ± 0.139; perfume = 0.462 ± 0.266; pine = 0.362 ± 0.194). Poorer fits (R^2^ < 0.2) were observed in 10 out of 76 functions, all of which occurred in nonfood blocks. Based on the results from the mixed effects regression model, different slopes were expected for food and nonfood blocks, and we thus retained all curves to accurately reflect participants’ performance (Fig. 4a). Results revealed no statistically significant differences in PSE (Fig. 4b) between any of the 4 target blocks (all p_adj_ > 0.1; individual test results in Supplementary Appendix G), indicating that the concentrations at which targets were perceived as dominant did not vary significantly between the odors (Table 5). In contrast, analysis of *JNDs* (Fig. 4c) showed statistically significant differences for all block comparisons (all p_adj_ ≤ 0.01; individual test results in Supplementary Appendix G), except for comparisons within the same target category (perfume vs. pine, broth vs. cake; both p_adj_ = 0.79). This demonstrated better perceptual separation of 2 food odors, independently of the attended odor.

**Table 5:** Descriptives of parameters from the psychometric function.

	Food target				Nonfood target			
	Broth		Cake		Perfume		Pine	
	Mdn	IQR	Mdn	IQR	Mdn	IQR	Mdn	IQR
PSE	51.869	13.312	48.203	16.769	53.273	33.312	51.828	25.192
JND	8.768	13.095	8.768	9.111	15.935	22.262	22.167	15.945

### 4.4. Exploratory analysis of attention

We explored whether attention modulated perceptual dominance by investigating the summed proportion of “yes” responses for physically identical odor mixtures across attentional blocks. This analysis mirrored the differential processing of food and nonfood dilution series demonstrated by all previous analyses. For food odors, the summed proportion significantly deviated from 1 at 0% (Mdn = 1.000, IQR = 0.107; M ± SD = 0.955 ± 0.081; V = 0, *P* = 0.031) and at 50% (Mdn = 1.000, IQR = 0.393, M ± SD = 0.887 ± 0.303; V = 29, *P* = 0.046). In both cases, the mean was below 1.0, suggesting that attention attenuated the perceived dominance of the target odor. For nonfood odors, no mixture level reached statistical significance (all *P* > 0.05), though a marginal effect was observed at 75% (Mdn = 0.929, IQR = 0.286; M ± SD = 0.870 ± 0.350; V = 32.5, *P* = 0.070), again hinting at possible attentional suppression. These results suggest a category-specific difference in how attention influences perceptual judgments, with food odors showing more consistent signs of attentional modulation around ambiguous mixtures (H3).

### 4.5. Physiological data: respiration

The number of inhalations correlated strongly with RT (r = 0.821, *P* < 0.001; Fig. 3e-f) and was negatively associated with response behavior (r = −0.055, *P* = 0.004) but showed no reliable associations with pleasantness or intensity ratings (Supplementary Appendix H).

## 5. Discussion

In this study, we investigated the effect of an attentional focus manipulation on the perception of ambivalent odor mixtures of 2 components. Our findings replicate previous research regarding the impact of odor concentration on decision-making and response time (Bowman et al. 2012; Shanahan et al. 2021; Seidel et al. 2025). Specifically, results showed a significant increase in the probability of perceiving the target odor as dominant as its concentration in the mixture increased (H1) and longer RT for more difficult odor mixtures compared to pure odors (H2). These findings demonstrate that participants were able to differentiate the 5 mixture steps of the 2 odor dilution series and that decision-making was easier for pure odors. Interestingly, results also showed that participants distinguished between the 2 food odors better than between the 2 nonfood odors, which may highlight the behavioral importance of food.

### 5.1. Food and nonfood odor mixtures are processed differently

By employing an odor decision-making task in 2 separate dilution series, we were able to assess how the effect of guided attention differentially influenced the perception of food and nonfood odor mixtures. We found that participants spent significantly longer time on decisions for nonfood than food odors, aligning with prior research in olfaction, which shows that food odors are detected more quickly than nonfood odors (Shanahan et al. 2021). This faster detection occurs regardless of the pleasantness of the stimulus (Boesveldt et al. 2010) and whether the food odor was attended to or not (Seidel et al. 2025). One explanation for this phenomenon is that food stimuli are recognized more easily due to their natural significance. In fact, faster processing has previously been observed in the visual domain, where participants react more quickly to food than nonfood pictures (Nummenmaa et al. 2011; Sawada et al. 2017; Mas et al. 2019). The current study supports the idea that detecting food targets is easier, demonstrated by the strong correlation between the number of inhalations and response time: participants were not merely thinking longer for more difficult trials and nonfood targets, but they were also smelling the odor more often. As such, considering inhalations to be instances of sampling evidence becomes a useful framework for thinking about task difficulty. In a previous study, Bowman et al. (2012) employed a 2-alternative categorization task on binary odor mixtures and found slower responses for more ambivalent odors as well as increased discrimination accuracy with increasing number of sniffs. In the current study, several participants spontaneously remarked that they found the task easier to complete for food than nonfood odors, and this is mirrored in the longer RT and higher number of sniffs seen for nonfood dilution series.

A similar distinction regarding the processing of food and nonfood odor mixtures was also evident in participants’ response behavior. Psychometric response curves for the 2 dilution series had different shapes, with food odors following a sigmoidal shape along the target odor concentration continuum, and nonfood odors showing a more linear increase. There was also a stark contrast in the 2 curves’ endpoints: participants were much more likely to incorrectly call a nonfood target odor dominant in a mixture that did not contain the target at all, than they were to do the same for a food target. On the flipside, when the target was presented in its pure form, participants were less likely to perceive it as dominant when it was a nonfood odor. This may suggest that acquiring a clear representation of an object is more difficult for a nonfood odor, or that participants may have needed more presentations of the pure nonfood targets prior to the decision-making task. These behavioral patterns were reflected in a significant simple effect of target and an interaction of target and its concentration. Across the dilution series spectrum, we observed a flatter increase in perceived target dominance as a result of increased target concentration for nonfood odors compared to foods, indicating better ability to separate between the 2 target stimuli in the food compared to the nonfood condition. These findings were also mirrored in the analyses of psychometric parameters extracted from the fitted response curves. Inspection of PSEs across the 4 target odors revealed that the concentration at which they were perceived as dominant on half of the trials did not vary statistically and was approximately 50%. On the other hand, JNDs were substantially lower for food targets, again highlighting that participants’ perception of food odor mixtures rapidly switched from target to nontarget, compared to that of nonfood mixtures.

These findings can be viewed in light of the well-documented theoretical distinction between configural and elemental processing, and the idea that food cues hold greater biological relevance. All of our target odors were real-world odor mixtures, rather than monomolecular volatile chemicals, so that stimuli in the odor dilution series became complex mixtures of many components. In this context, our findings may suggest that the observed differences between food and nonfood targets may be driven by a greater reliance on configural processing for food odors. Recognition of an odor as a familiar food object enables both efficient separation from a noisy odor background and perceptual stability across different contexts (Gottfried 2010; Stevenson 2014). Such configural processing could also facilitate the identification of food odors, even when presented in an odor mixture with another food object, thereby reducing decision difficulty. Steep psychometric functions for food targets would thereby indicate rapid shifts in perception once a threshold concentration was reached: an attended cake target was perceived as cake-dominant until it suddenly was not. This is in line with the concept of pattern separation, where 2 olfactory inputs to the system are more likely to be perceived as separate objects as their cortical activation patterns become more dissimilar (Barnes et al. 2008; Wilson 2009; Gottfried 2010; Wilson and Sullivan 2011).

A cortically stored odor object is likely more defined and robust for food-related odors than for nonfood odors. Food odors, such as cake or broth, are usually experienced in a multisensory fashion, where olfactory, gustatory, and somatosensory inputs converge during consumption. This repeated co-activation strengthens multisensory representations in areas such as frontal operculum, parts of the insula, the caudal orbitofrontal cortex, and the amygdala (de Araujo et al. 2003; Small et al. 2004; Small and Prescott 2005; Veldhuizen et al. 2010; Khorisantono et al. 2025), supporting more stable odor templates. In contrast, nonfood odors like pine or perfume, which lack such rich associations, may be represented more broadly. It has been suggested that odors are perceived in an object-based manner based on the functional goals they are associated with (Gottfried 2009; Stevenson 2014); as such, food odor objects should be dependent on the goal of potential ingestion. Because consuming spoiled or unfamiliar food poses a significant risk, organisms should be more conservative when evaluating potential contamination of a familiar food odor compared to a noningestible odor. This enhanced sensitivity to deviations in food-related olfactory signals (i.e. higher contamination sensitivity) could underlie the clearer separation observed for food targets in the current study. Such reasoning is also consistent with findings that, compared to visual tasks, detecting differences between odors tends to be faster than detecting sameness (Møller et al. 2012; Köster et al. 2014). If the internal representation of a “cake” odor is more narrowly defined, less tolerant to variation, than that of a “pine” odor, deviations from this template should be detected more rapidly, as also reflected in the response time data of the current study.

### 5.2. Attention affects perception only for some levels of the food odor mixtures

While these findings point to inherently sharper representations for food odors the extent to which top-down attention can modulate such perceptual boundaries remains speculative. In the current study, participants completed the perceptual decision-making task twice for each odor dilution series. By changing the target odor within the dilution series between blocks we aimed to determine whether attending to an odor component would alter its perceived dominance within the mixtures. Top-down selective attention may facilitate goal-directed behavior through the selective processing of certain relevant features in the environment (Veldhuizen 2017). In line with this, one might anticipate perceptual enhancement of attended odors compared to background odors of low task relevance. Indeed, there is previous research indicating that the anterior piriform cortex and the olfactory tubercle will show increased activation when anticipating an odor rather than clean air, or when anticipating an odor that is relevant to the task at hand (Zelano et al. 2005). Moreover, it has been suggested that detection is improved for attended odors. More specifically, Zelano et al. (2011) demonstrated that top-down input from orbitofrontal areas, in combination with bottom-up input from the piriform cortex, may support a predictive coding mechanism via the mediodorsal thalamus. This system allows for the comparison of expected and incoming sensory information, thereby guiding perception in a goal-relevant manner.

Our behavioral data, however, showed limited evidence for such attentional modulation. On a general level, there were no statistical differences in psychometric parameters, JND and PSE, between targets within the same dilution series. As such, no odor became generally more dominant when attended, and neither did attention significantly change perceptual separation of target and nontarget odors. To gain a more nuanced understanding of how attentional focus may have influenced behavior, we investigated the proportion of perceived target dominance for the same physical stimulus over the 2 blocks with different attentional foci. Contrary to the theoretical mechanism outlined above, the attended odor was never perceptually amplified (H3). Moreover, attention did not influence perception at all for the nonfood dilution series. However, among the food odors, there was some indication for attentional effects in the most ambivalent stimulus (50% target odor concentration) and when the target odor was completely absent (0% target odor concentration). Interestingly, in both instances, the attended odor was perceptually attenuated. Since this finding is based on an exploratory analysis in a set of only 2 odors, future research should try to replicate this outcome. One possible explanation for this pattern is that attention may only influence perception when there is a high level of ambiguity. In the case of food odors, participants seem to adopt a more conservative decision-making strategy. This aligns with our previous findings, which indicate that food targets generally require higher concentrations to achieve perceptual dominance compared to nonfood targets (Seidel et al. 2025). In this context, attention may not serve to amplify the target’s perceptual presence but instead help participants apply stricter evaluative criteria for biologically relevant stimuli. This approach would be adaptive for food odors, as misclassifying a potentially contaminated or unfamiliar item could pose a risk. In contrast, for nonfood odors, which are less relevant to ingestion and survival, attentional focus did not show any measurable effects (H4).

### 5.3. Limitations

The current study has some limitations that should be discussed. First, while we chose to only include odors that represented familiar objects and introduced each of them along with a picture to enhance object association, we did not explicitly assess familiarity. In the absence of such data, we cannot conclusively rule out that higher everyday exposure to food odors may have contributed to the observed sharper perceptual boundaries for food odors. It is also possible that other odors with strong evolutionary significance, such as social odors or danger-related nonfood odors, would produce similar effects. While it may seem unlikely that the specific odors of broth and cake used in this research would be more recognizable than the nonfood odors, future studies could enhance their findings by conducting a more thorough evaluation of how participants rated the stimulus materials based on aspects such as familiarity.

The present study only used 1 odor pair per category. As a result, category-level effects (food vs. nonfood) cannot be fully disentangled from stimulus-specific properties such as perceptual similarity, complexity, or potential overlap in molecular composition. Although the stimuli were carefully selected and piloted to be perceptually distinguishable and matched on key dimensions, it remains possible that differences in discriminability between the specific odor pairs contributed to the observed effects. Future work should include multiple stimulus exemplars per category and more controlled manipulation of perceptual similarity and task difficulty to more clearly separate category-level from stimulus-level influences. Future studies could also employ more chemically defined or monomolecular odorants to increase experimental control over mixture composition and perceptual overlap, although such approaches may involve reduced ecological validity and recognizability of odor objects.

Moreover, while we paired odor targets with visual representations of their sources to aid classification into food and nonfood items, we did not formally assess the perceived edibility of the stimuli. Importantly, we did demonstrate that differences in separability seen between food and nonfood odors are unlikely to stem from differences in pleasantness or intensity of the stimulus material. Throughout the experiment, there were no significant systematic changes in either pleasantness or intensity ratings over time, nor any evidence of baseline differences in intensity or pleasantness between the odors. We therefore deem adaptation an unlikely influence on decision behavior.

Fourth, the absence of a neutral baseline condition without directed attention limits the interpretation of the attentional effects. Although we observed a reduction in the perception of food odors under certain conditions, our analysis relied solely on “yes” responses. As such, a “no” response cannot distinguish between dominance of the nontarget odor or a state of perceptual ambiguity where both components are perceived as equally present. Along the same line of reasoning, we do not know how participants handled situations in which they were uncertain, and it should be acknowledged that response biases are common concerns for forced-choice tasks (García-Pérez and Alcalá-Quintana 2013). Incorporating certainty ratings in future studies could help to capture these nuances, but this approach would also substantially increase task duration, reducing the number of repetitions per stimulus and, consequently, the reliability of parameter estimation. Including a no-attention baseline condition would further help to isolate the true influence of attentional focus.

Finally, our sample size may have limited the ability to reliably detect small effects, particularly higher-order interactions. Accordingly, null findings should be interpreted with caution.

### 5.4. Conclusion

In summary, our study indicates that perceptual boundaries between odors are sharper for food than for nonfood odors, suggesting that food-related olfactory information is processed differently and with greater perceptual conservatism. This pattern may reflect the significance of accurately identifying potentially ingestible stimuli. While we found limited evidence for a general effect of an attentional focus manipulation, exploratory results point to the possibility that attention may perceptually attenuate a food odor target when uncertainty is high. Together, these results advance our understanding of how the perceptual system balances sensitivity and caution in olfactory decision-making and provide a behavioral basis for future work linking these processes to their underlying neural mechanisms.

### Lead contact

Further information and requests for resources and reagents should be directed to and will be fulfilled by the lead contact, Leonie Seidel (leonie.seidel@ki.se).

## Supplementary Material

bjag021_Supplementary_Data

## Data Availability

Data will be made available on the associated OSF project upon publication.

## References

[bjag021-B1] Albrecht J, Schreder T, Kleemann AM, Schöpf V, Kopietz R, Anzinger A, Demmel M, Linn J, Kettenmann B, Wiesmann M. Olfactory detection thresholds and pleasantness of a food-related and a non-food odour in hunger and satiety. Rhinology. 2009:47(2):160–165. 19593973

[bjag021-B2] Barnes DC, Hofacer RD, Zaman AR, Rennaker RL, Wilson DA. Olfactory perceptual stability and discrimination. Nat Neurosci. 2008:11(12):1378–1380. 10.1038/nn.2217 18978781 PMC2682180

[bjag021-B3] Bates D, Mächler M, Bolker B, Walker S. Fitting linear mixed-effects models using lme4. J Stat Softw. 2015:67(1):1–48. 10.18637/jss.v067.i01

[bjag021-B4] Blechert J, Lender A, Polk S, Busch NA, Ohla K. Food-pics_extended-an image database for experimental research on eating and appetite: additional images, normative ratings and an updated review. Front Psychol. 2019:10:307. 10.3389/fpsyg.2019.00307 30899232 PMC6416180

[bjag021-B5] Boesveldt S, Frasnelli J, Gordon AR, Lundström JN. The fish is bad: negative food odors elicit faster and more accurate reactions than other odors. Biol Psychol. 2010:84(2):313–317. 10.1016/j.biopsycho.2010.03.006 20227457

[bjag021-B6] Bowman NE, Kording KP, Gottfried JA. Temporal integration of olfactory perceptual evidence in human orbitofrontal cortex. Neuron. 2012:75(5):916–927. 10.1016/j.neuron.2012.06.035 22958830 PMC3441053

[bjag021-B7] Coureaud G, Thomas-Danguin T, Sandoz J-C, Wilson DA. Biological constraints on configural odour mixture perception. J Exp Biol. 2022:225(6):jeb242274. 10.1242/jeb.242274 35285471 PMC8996812

[bjag021-B8] de Araujo IE, Rolls ET, Kringelbach ML, McGlone F, Phillips N. Taste-olfactory convergence, and the representation of the pleasantness of flavour, in the human brain. Eur J Neurosci. 2003:18(7):2059–2068. 10.1046/j.1460-9568.2003.02915.x 14622239

[bjag021-B9] Fontana L, Albayay J, Fernández-Prieto I, Zampini M. Olfactory habituation to food and nonfood odours. Q J Exp Psychol. 2023:76(6):1209–1219. 10.1177/17470218221115046 35866345

[bjag021-B10] Freeman WJ . A physiological hypothesis of perception. Perspect Biol Med. 1981:24(4):561–592. 10.1353/pbm.1981.0036 7027172

[bjag021-B11] Freeman WJ . The physiological basis of mental images. Biol Psychiatry. 1983:18(10):1107–1125. 6317064

[bjag021-B12] García-Pérez MA, Alcalá-Quintana R. Shifts of the psychometric function: distinguishing bias from perceptual effects. Q J Exp Psychol. 2013:66(2):319–337. 10.1080/17470218.2012.708761 22950887

[bjag021-B13] Gescheider GA . Psychophysics: the fundamentals. 3rd ed. Lawrence Erlbaum Associates Publishers; 1997. 10.4324/9780203774458

[bjag021-B14] Ghibaudo V, Granget J, Dereli M, Buonviso N, Garcia S. A unifying method to study respiratory sinus arrhythmia dynamics implemented in a new toolbox. ENeuro. 2023:10(10):ENEURO.0197-23.2023. 10.1523/ENEURO.0197-23.2023 PMC10614108 37848290

[bjag021-B15] Gottfried JA . Function follows form: ecological constraints on odor codes and olfactory percepts. Curr Opin Neurobiol. 2009:19(4):422–429. 10.1016/j.conb.2009.07.012 19671493 PMC2761641

[bjag021-B16] Gottfried JA . Central mechanisms of odour object perception. Nat Rev Neurosci. 2010:11(9):628–641. 10.1038/nrn2883 20700142 PMC3722866

[bjag021-B17] Howard JD, Plailly J, Grueschow M, Haynes J-D, Gottfried JA. Odor quality coding and categorization in human posterior piriform cortex. Nat Neurosci. 2009:12(7):932–938. 10.1038/nn.2324 19483688 PMC2834563

[bjag021-B18] Hummel T, Sekinger B, Wolf SR, Pauli E, Kobal G. “Sniffin” sticks’: olfactory performance assessed by the combined testing of odor identification, odor discrimination and olfactory threshold. Chem Senses. 1997:22(1):39–52. 10.1093/chemse/22.1.39 9056084

[bjag021-B19] Jinks A, Laing DG. A limit in the processing of components in odour mixtures. Perception. 1999:28(3):395–404. 10.1068/p2898 10615476

[bjag021-B20] Kay LM, Crk T, Thorngate J. A redefinition of odor mixture quality. Behav Neurosci. 2005:119(3):726–733. 10.1037/0735-7044.119.3.726 15998193

[bjag021-B21] Khorisantono PA, Veldhuizen MG, Seubert J. Tastes and retronasal odours evoke a shared flavour-specific neural code in the human insula. BioRxiv 2025.01.06.631354. 2025. 10.1101/2025.01.06.631354 PMC12432251 40940344

[bjag021-B22] Kingdom F, Prins N. Psychophysics: a practical introduction. 2nd ed. Academic Press; 2016, p. 1–331.

[bjag021-B23] Knoblauch K, Maloney LT. Modeling psychophysical data in R. New York: Springer; 2012. 10.1007/978-1-4614-4475-6

[bjag021-B24] Köster EP . Does olfactory memory depend on remembering odors? Chem Senses. 2005:30(Supplement 1):i236–i237. 10.1093/chemse/bjh201 15738133

[bjag021-B25] Köster EP, Møller P, Mojet J. A “Misfit” theory of spontaneous conscious odor perception (MITSCOP): reflections on the role and function of odor memory in everyday life. Front Psychol. 2014:5:64. 10.3389/fpsyg.2014.00064 24575059 PMC3920064

[bjag021-B26] Laing DG, Francis GW. The capacity of humans to identify odors in mixtures. Physiol Behav. 1989:46(5):809–814. 10.1016/0031-9384(89)90041-3 2628992

[bjag021-B27] Lakens D . Equivalence tests: a practical primer for t tests, correlations, and meta-analyses. Soc Psychol Personal Sci. 2017:8(4):355–362. 10.1177/1948550617697177 28736600 PMC5502906

[bjag021-B28] Lakens D, Scheel AM, Isager PM. Equivalence testing for psychological research: a tutorial. Adv Methods Pract Psychol Sci. 2018:1(2):259–269. 10.1177/2515245918770963

[bjag021-B29] Livermore A, Laing DG. The influence of odor type on the discrimination and identification of odorants in multicomponent odor mixtures. Physiol Behav. 1998:65(2):311–320. 10.1016/s0031-9384(98)00168-1 9855481

[bjag021-B30] Lundström JN, Gordon AR, Alden EC, Boesveldt S, Albrecht J. Methods for building an inexpensive computer-controlled olfactometer for temporally-precise experiments. Int J Psychophysiol. 2010:78(2):179–189. 10.1016/j.ijpsycho.2010.07.007 20688109 PMC2967213

[bjag021-B31] Mas M, Brindisi M-C, Chabanet C, Nicklaus S, Chambaron S. Weight status and attentional biases toward foods: impact of implicit olfactory priming. Front Psychol. 2019:10:1789. 10.3389/fpsyg.2019.01789 31447733 PMC6696981

[bjag021-B32] Maurer D, Grand RL, Mondloch CJ. The many faces of configural processing. Trends Cogn Sci. 2002:6(6):255–260. 10.1016/S1364-6613(02)01903-4 12039607

[bjag021-B33] McCarley JS, Yamani Y. Psychometric curves reveal three mechanisms of vigilance decrement. Psychol Sci. 2021:32(10):1675–1683. 10.1177/09567976211007559 34543100

[bjag021-B34] Mcfadden D , 1977. Quantitative methods for analyzing travel behaviour of individuals: some recent developments. Cowles Foundation Discussion Papers 474, Cowles Foundation for Research in Economics, Yale University.

[bjag021-B35] Meteyard L, Davies RAI. Best practice guidance for linear mixed-effects models in psychological science. J Mem Lang. 2020:112:104092. 10.1016/j.jml.2020.104092

[bjag021-B36] Miller SS, Gordon AR, Olsson MJ, Lundström JN, Dalton P. Mind over age–stereotype activation and olfactory function. Chem Senses. 2013:38(2):167–174. 10.1093/chemse/bjs086 23118205

[bjag021-B37] Møller P, Köster EP, Dijkman N, de Wijk R, Mojet J. Same–different reaction times to odors: some unexpected findings. Chemosens Percept. 2012:5(2):158–171. 10.1007/s12078-012-9124-x

[bjag021-B38] Nie H, Zhao R, Ai Y, Yang Y, Cao B, Han P. Comparison between human olfactory sensitivity in the fasted and fed states: a systematic review and meta-analysis. Appetite. 2023:181:106395. 10.1016/j.appet.2022.106395 36450324

[bjag021-B39] Nummenmaa L, Hietanen JK, Calvo MG, Hyönä J. Food catches the eye but not for everyone: a BMI-contingent attentional bias in rapid detection of nutriments. PLoS One. 2011:6(5):e19215. 10.1371/journal.pone.0019215 21603657 PMC3095600

[bjag021-B40] Peirce J, Gray JR, Simpson S, MacAskill M, Höchenberger R, Sogo H, Kastman E, Lindeløv JK. Psychopy2: experiments in behavior made easy. Behav Res Methods. 2019:51(1):195–203. 10.3758/s13428-018-01193-y 30734206 PMC6420413

[bjag021-B41] Plailly J, Howard JD, Gitelman DR, Gottfried JA. Attention to odor modulates thalamocortical connectivity in the human brain. J Neurosci. 2008:28(20):5257–5267. 10.1523/JNEUROSCI.5607-07.2008 18480282 PMC2706104

[bjag021-B42] Poupon D, Fernandez P, Archambault Boisvert S, Migneault-Bouchard C, Frasnelli J. Can the identification of odorants within a mixture be trained? Chem Senses. 2018:43(9):721–726. 10.1093/chemse/bjy060 30260369

[bjag021-B43] Rossion B . The composite face illusion: a whole window into our understanding of holistic face perception. Vis Cogn. 2013:21(2):139–253. 10.1080/13506285.2013.772929

[bjag021-B44] Savva A, Dijkman R, Bulik CM, Seubert J. Behavioral separation of liking and wanting in response to olfactory and visual food cues. Appetite. 2025:204:107717. 10.1016/j.appet.2024.107717 39423862

[bjag021-B45] Sawada R, Sato W, Toichi M, Fushiki T. Fat content modulates rapid detection of food: a visual search study using fast food and Japanese diet. Front Psychol. 2017:8:1033. 10.3389/fpsyg.2017.01033 28690568 PMC5479904

[bjag021-B46] Schaefer M, Hrysanidis C, Lundström JN, Arshamian A. Phase-locked breathing does not affect episodic visual recognition memory but does shape its corresponding ERPs. Psychophysiology. 2024:61(4):e14493. 10.1111/psyp.14493 38053412

[bjag021-B47] Seidel L, Kilteni K, Lundström JN, Seubert J. Sensitivity to contamination of food odours depends on hunger and attention. Brain Res. 2025:1864:149812. 10.1016/j.brainres.2025.149812 40614797

[bjag021-B48] Seubert J, Ohla K, Yokomukai Y, Kellermann T, Lundström JN. Superadditive opercular activation to food flavor is mediated by enhanced temporal and limbic coupling. Hum Brain Mapp. 2015:36(5):1662–1676. 10.1002/hbm.22728 25545699 PMC6868967

[bjag021-B49] Shanahan LK, Bhutani S, Kahnt T. Olfactory perceptual decision-making is biased by motivational state. PLoS Biol. 2021:19(8):e3001374. 10.1371/journal.pbio.3001374 34437533 PMC8389475

[bjag021-B50] Shanahan LK, Kahnt T. On the state-dependent nature of odor perception. Front Neurosci. 2022:16:964742. 10.3389/fnins.2022.964742 36090268 PMC9459319

[bjag021-B51] Small DM, Prescott J. Odor/taste integration and the perception of flavor. Exp Brain Res. 2005:166(3–4):345–357. 10.1007/s00221-005-2376-9 16028032

[bjag021-B52] Small DM, Voss J, Mak YE, Simmons KB, Parrish T, Gitelman D. Experience-dependent neural integration of taste and smell in the human brain. J Neurophysiol. 2004:92(3):1892–1903. 10.1152/jn.00050.2004 15102894

[bjag021-B53] Sorokowski P, Karwowski M, Misiak M, Marczak MK, Dziekan M, Hummel T, Sorokowska A. Sex differences in human olfaction: a meta-analysis. Front Psychol. 2019:10:242. 10.3389/fpsyg.2019.00242 30814965 PMC6381007

[bjag021-B54] Spence C, McGlone F, Kettenmann B, Kobal G. Attention to olfaction. A psychophysical investigation. Exp Brain Res. 2001:138(4):432–437. 10.1007/s002210100713 11465740

[bjag021-B55] Stevenson RJ . Object concepts in the chemical senses. Cogn Sci. 2014:38(7):1360–1383. 10.1111/cogs.12111 24641582

[bjag021-B56] Stewart MG, Witsell DL, Smith TL, Weaver EM, Yueh B, Hannley MT. Development and validation of the Nasal Obstruction Symptom Evaluation (NOSE) scale. Otolaryngol-Head Neck Surg. 2004:130(2):157–163. 14990910 10.1016/j.otohns.2003.09.016

[bjag021-B57] Taubert J, Apthorp D, Aagten-Murphy D, Alais D. The role of holistic processing in face perception: evidence from the face inversion effect. Vis Res. 2011:51(11):1273–1278. 10.1016/j.visres.2011.04.002 21496463

[bjag021-B58] Thomas-Danguin T, Sinding C, Romagny S, El Mountassir F, Atanasova B, Le Berre E, Le Bon A-M, Coureaud G. The perception of odor objects in everyday life: a review on the processing of odor mixtures. Front Psychol. 2014:5:504. 10.3389/fpsyg.2014.00504 24917831 PMC4040494

[bjag021-B59] Toet A, Kaneko D, de Kruijf I, Ushiama S, van Schaik MG, Brouwer A-M, Kallen V, van Erp JBF. CROCUFID: a cross-cultural food image database for research on food elicited affective responses. Front Psychol. 2019:10:58. 10.3389/fpsyg.2019.00058 30740078 PMC6355693

[bjag021-B60] Veldhuizen MG . Distracted sniffing of food odors leads to diminished behavioral and neural responses. Chem Senses. 2017:42(9):719–722. 10.1093/chemse/bjx060 28968700 PMC5863562

[bjag021-B61] Veldhuizen MG, Nachtigal D, Teulings L, Gitelman DR, Small DM. The insular taste cortex contributes to odor quality coding. Front Hum Neurosci. 2010:4:58. 10.3389/fnhum.2010.00058 20700500 PMC2917218

[bjag021-B62] Veldhuizen MG, Small DM. Modality-specific neural effects of selective attention to taste and odor. Chem Senses. 2011:36(8):747–760. 10.1093/chemse/bjr043 21685407 PMC3175104

[bjag021-B63] Vennemann MM, Hummel T, Berger K. The association between smoking and smell and taste impairment in the general population. J Neurol. 2008:255(8):1121–1126. 10.1007/s00415-008-0807-9 18677645

[bjag021-B64] Wilson DA . Pattern separation and completion in olfaction. Ann N Y Acad Sci. 2009:1170(1):306–312. 10.1111/j.1749-6632.2009.04017.x 19686152 PMC2771856

[bjag021-B65] Wilson DA, Fleming G, Vervoordt SM, Coureaud G. Cortical processing of configurally perceived odor mixtures. Brain Res. 2020:1729:146617. 10.1016/j.brainres.2019.146617 31866364 PMC6941848

[bjag021-B66] Wilson DA, Sullivan RM. Cortical processing of odor objects. Neuron. 2011:72(4):506–519. 10.1016/j.neuron.2011.10.027 22099455 PMC3223720

[bjag021-B67] Zelano C, Bensafi M, Porter J, Mainland J, Johnson B, Bremner E, Telles C, Khan R, Sobel N. Attentional modulation in human primary olfactory cortex. Nat Neurosci. 2005:8(1):114–120. 10.1038/nn1368 15608635

[bjag021-B68] Zelano C, Mohanty A, Gottfried JA. Olfactory predictive codes and stimulus templates in piriform cortex. Neuron. 2011:72(1):178–187. 10.1016/j.neuron.2011.08.010 21982378 PMC3190127

