# Supplementary material for: Attentional modulation of contamination sensitivity: sharper perceptual boundaries for food compared to nonfood odor mixtures
Source: Chem Senses. 2026 Jul 28;51:bjag021. doi: 10.1093/chemse/bjag021 (PMC13547862; doi:10.1093/chemse/bjag021)
Supplement: Supplementary file 1 [file bjag021_supplementary_data.docx]

**Supplemental Materials**

**Contamination Sensitivity in complex Odour Mixtures: Sharper Perceptual Boundaries for Attended Food compared to Non-Food Odours**

Leonie Seidel^1,2*^, Astrid Endrup Iversen^3^, Konstantina Kilteni^4,5^, Janina Seubert^1*^

^1^ Department of Clinical Neuroscience, Karolinska Institutet, Stockholm, Sweden

^2^ Lead contact

^3^ Department of Psychology, Copenhagen University, Copenhagen, Denmark

^4^ Donders Institute for Brain, Cognition & Behaviour, Nijmegen, The Netherlands

^5^ Department of Neuroscience, Karolinska Institutet, Stockholm, Sweden

*Correspondence: leonie.seidel@ki.se

**Correspondence: janina.seubert@ki.se

Index:

**Appendix A**, *Materials availability*

**Appendix B**, *Model comparisons (response)*

**Appendix C**, *Model comparisons (response time)*

**Appendix D**, *Optimiser checks*

**Appendix E**, *Equivalence testing*

**Appendix F**, *Random effects*

**Appendix G**, *Psychometric parameters*

**Appendix H**, *Respiration*

**Appendix A**

*Materials availability*

| RESOURCE | SOURCE | IDENTIFIER |
| --- | --- | --- |
| Software and algorithms | | |
| R Version 4.4.3 | R Core Team (2024) | https://www.r-project.org/ |
| R Studio Version 2025.05.0 | Posit Team | https://posit.co/ |
| Other | | |
| Vegetable Broth Odour | International Flavors and Fragrances (Mirepoix) | https://www.iff.com/ |
| Cake Odour | Stockholms Aeter & Essencefabrik (Cake Essence för smaksättning) | https://www.essencefabriken.se/sortiment.htm |
| Watermelon Odour | Stockholms Aeter & Essencefabrik (Vattenmelon Essence för smaksättning) | https://www.essencefabriken.se/sortiment.htm |
| Lavender Odour | Stockholms Aeter & Essencefabrik (Lavendel Eteriska oljor/Parfymoljor) | https://www.essencefabriken.se/sortiment.htm |
| Perfume Odour | Dr. Vranjes Firenze (Ambra) | https://drvranjes.com/eu/ambra-home-fragrance |
| Pine Odour | Essences Bulgaria (Abies alba essential oil) | https://essences-bulgaria.com/item/1388 |
| Vegetable Broth Picture | Adapted from Unsplash (photograph by [Monika Grabkowska](https://unsplash.com/@moniqa)) | https://unsplash.com/photos/several-vegetable-dishes-9jdyCy-bMC4 |
| Cake Picture | CROCUFID food image database | Toet, A., Kaneko, D., De Kruijf, I., Ushiama, S., Van Schaik, M. G., Brouwer, A. M., ... & Van Erp, J. B. (2019). CROCUFID: A cross-cultural food image database for research on food elicited affective responses. *Frontiers in psychology*, *10*, 58. |
| Watermelon Picture | CROCUFID food image database | Toet, A., Kaneko, D., De Kruijf, I., Ushiama, S., Van Schaik, M. G., Brouwer, A. M., ... & Van Erp, J. B. (2019). CROCUFID: A cross-cultural food image database for research on food elicited affective responses. *Frontiers in psychology*, *10*, 58. |
| Lavender Picture | Adapted from Unsplash (photograph by [Olga Tutunaru](https://unsplash.com/@otutunaru)) | https://unsplash.com/photos/white-flowers-with-green-leaves-B9hX9HnTm2Q |
| Perfume Picture | Adapted from Unsplash (photograph by [Fernando Andrade](https://unsplash.com/@thisisnando)) | https://unsplash.com/photos/n5-chanel-eau-de-parfum-spray-bottle-potCPE_Cw8A |
| Pine Picture | Food-Pics_Extended Database | Blechert, J., Lender, A., Polk, S., Busch, N. A., & Ohla, K. (2019). Food-pics_extended—an image database for experimental research on eating and appetite: additional images, normative ratings and an updated review. *Frontiers in psychology*, *10*, 307. |
| PowerLab C (product code PLC01) | ADInstruments | https://www.adinstruments.com/products/powerlab/c?srsltid=AfmBOorIRTJ7N4pPJA_bIon4n92qdzop9SdrRotunU30hAHB1-sccW-H |
| C-Series Instrument Interface (product code PlCI1) | ADInstruments | https://www.adinstruments.com/products/instrument-interface?srsltid=AfmBOopKQD6MFHMzI3T21w4RW1zynJwgNmTFf9h8OAZBcJlpJH5bplAb |
| Nasal Temperature Probe (product code MLT415) | ADInstruments | https://www.adinstruments.com/products/nasal-temperature-probes?srsltid=AfmBOoo4CIZ-fMiIZVQWl3cngotzRXAKbtfIrTx28HZDJfcx-IC_zsVh |
| Respiratory Belt Transducer (product code TN1132/ST) | ADInstruments | https://www.adinstruments.com/products/respiratory-belt-transducer?srsltid=AfmBOorEY5pudmE1IvAP7jBxSVaxOV3F1ooQsK6K3JI0m5NUtsJQTLaq |

**Appendix B**

*Model comparisons (response)*

As recommended in the best practice guidelines by [Meteyard & Davies (2020)](https://sciwheel.com/work/citation?ids=8174072&pre=&suf=&sa=0&dbf=1)

- We explored structure systematically (random structure, then fixed structure); Models 1-5
- Reviewer feedback triggered an additional targeted test; Models 6 and 7
- Model 7 is the final reported model

| Sampling Units | N total obs = 3077; N Subjects = 22 | | | | | | |
| --- | --- | --- | --- | --- | --- | --- | --- |
| **Model specification**  glmerControl(optimizer = "bobyqa", optCtrl = list(maxfun = 20000)) | **Random Effects** | **Model fit** | | | | **LRT Test against previous** | |
|  |  | **AIC** | **BIC** | **LogLik** | **npar** | **df** | **Χ^2^** |
| **Model 1**  Target concentration * target * gender + rt_z | (1+block\|subj_idx) + (1\|block) | 2470 | 2591 | -1215 | 20 |  |  |
| **Model 2**  Removed random effect | (1+block\|subj_idx) | 2468 | 2583 | -1215 | 19 | 1 | 0 |
| **Model 3**  Removed 3-way interaction:  target concentration × target × gender | (1+block\|subj_idx) | 2466 | 2575 | -1215 | 18 | 1 | 0.05 |
| **Model 4**  Removed 2-way interaction:  target concentration × gender | (1+block\|subj_idx) | 2465 | 2568 | -1216 | 17 | 1 | 0.92 |
| **Model 5**  Removed 2-way interaction:  target × gender | (1+block\|subj_idx) | 2465 | 2562 | -1217 | 16 | 1 | 1.9 |
| **Model 6**  Fixed effects constant from previous  Produced singularity | (1+block × target concentration\|subj_idx) |  |  |  |  |  |  |
| **Model 7**  Fixed effects constant from previous  Compared against Model 5 | (1+block + target concentration\|subj_idx) | 2393 | 2520 | -1176 | 21 | 5 | 81.9 |

**Appendix C**

*Model comparisons (response time)*

As recommended in the best practice guidelines by [Meteyard & Davies (2020)](https://sciwheel.com/work/citation?ids=8174072&pre=&suf=&sa=0&dbf=1)

- We explored structure systematically (random structure, then fixed structure); Models 1-9
- Reviewer feedback triggered an additional targeted test; Models 10 and 11
- Model 8 is the final reported model

| Sampling Units | N total obs = 3077; N Subjects = 22 | | | | | | |
| --- | --- | --- | --- | --- | --- | --- | --- |
| **Model specification**  lmerControl(optimizer = "nplotwrap", optCtrl = list(maxfun = 20000)) | **Random Effects** | **Model fit** | | | | **LRT Test against previous** | |
|  |  | **AIC** | **BIC** | **LogLik** | **npar** | **df** | **Χ^2^** |
| **Model 1**  Target concentration * target * gender +  target concentration^2^ * target * gender + response | (1+block\|subj_idx) + (1\|block) | 5022 | 5173 | -2486 | 25 |  |  |
| **Model 2**  Removed random intercept | (1+block\|subj_idx) | 5022 | 5167 | -2487 | 24 | 1 | 2.03 |
| **Model 3**  Removed 3-way interaction:  target concentration × target × gender | (1+block\|subj_idx) | 5022 | 5161 | -2488 | 23 | 1 | 1.53 |
| **Model 4**  Removed 3-way interaction:  target concentration^2^ × target × gender | (1+block\|subj_idx) | 5022 | 5154 | -2489 | 22 | 1 | 1.71 |
| **Model 5**  Removed 2-way interaction:  target concentration × gender | (1+block\|subj_idx) | 5020 | 5147 | -2489 | 21 | 1 | 0.13 |
| **Model 6**  Removed 2-way interaction:  target × target concentration^2^ | (1+block\|subj_idx) | 5018 | 5139 | -2489 | 20 | 1 | 0.18 |
| **Model 7**  Removed 2-way interaction:  target × gender | (1+block\|subj_idx) | 5016 | 5131 | -2489 | 19 | 1 | 0.27 |
| **Model 8**  Removed 2-way interaction:  target concentration^2^ × gender | (1+block\|subj_idx) | 5015 | 5124 | -2490 | 18 | 1 | 0.74 |
| **Model 9**  Removed 2-way interaction:  target concentration × target | (1+block\|subj_idx) | 5017 | 5119 | -2491 | 17 | 1 | 3.69 |
| **Model 10**  fixed effects constant from Model 7  Compared against Model 8 | (1+block + target concentration\|subj_idx) | 5085 | 5224 | -2519 | 23 | 5 | 0 |
| **Model 11**  fixed effects constant from previous  Compared against Model 8 | (1+block + target concentration\|subj_idx) | 5020 | 5159 | -2487 | 23 | 5 | 4.69 |

**Appendix D**

*Optimiser checks*

For the final models of both variables, optimiser checks were performed using the *allFit()* function, to ensure that our models’ parameters were not dependent on the chosen optimisers.

***Figure S1.***

**Appendix E**

*Equivalence testing*

| *Equivalence of Ratings within Odours (Pre- vs. Post-Task)* | | | |
| --- | --- | --- | --- |
|  | *Pleasantness* |  | *Intensity* |
| Broth | **Not equivalent** [t(23) = -0.76, p = 0.23] and **different** [t(23) = 2.16, p = 0.01]  Hedges' g(z) = [0.40, 0.89] |  | **Equivalent** [t(23) = 2.33, p = 0.02] and **not different** [t(23) = -0.59, p = 0.49]  Hedges' g(z) = [-0.30, -0.01] |
| Cake | **Equivalent** [t(23) = 2.5, p = 0.01] and **not different** [t(23) = -0.37, p = 0.73]  Hedges' g(z) = [-0.22, 0.02] |  | **Equivalent** [t(23) = -2.87, p = 0.03]  and **not different** [t(23) = 0.06, p = 0.90]  Hedges' g(z) = [-0.25, 0.41] |
| Perfume | **Equivalent** [t(23) = 2.56, p = 0.003] and **not different** [t(23) = -0.43, p = 0.78]  Hedges' g(z) = [-0.30, 0.13] |  | **Not equivalent** [t(23) = -1.22,  p = 0.10] and **not different** [t(23) = 1.71, p = 0.91]  Hedges' g(z) = [-0.05, 0.70] |
| Pine | **Equivalent** [t(23) = -1.77, p = 0.048] and **not different** [t(23) = 1.16, p = 0.21]  Hedges' g(z) = [0.18, 0.42] |  | **Equivalent** [t(23) = 2.54, p = 0.01] and **not different** [t(23) = -0.39, p = 0.67]  Hedges' g(z) = [0.40, 0.89] |

| *Equivalence of Ratings between Odours (Pre-Task)* | | | |
| --- | --- | --- | --- |
|  | *Pleasantness* |  | *Intensity* |
| Broth vs. Cake | **Equivalent** [t(23) = 1.86, p = 0.04]  and **not different** [t(23) = -1.06, p = 0.30]  Hedges' g(z) = [-0.66, 0.40] |  | **Equivalent** [t(23) = 2.01, p = 0.02]  and **not different** [t(23) = -0.92, p = 0.36]  Hedges' g(z) = [-0.47, 0.08] |
| Broth vs. Perfume | **Not equivalent** [t(23) = 1.46, p = 0.08] and **not different** [t(23) =  -1.46, p = 0.14]  Hedges' g(z) = [-0.75, 0.32] |  | **Not equivalent** [t(23) = 1.30, p = 0.09] and **not different** [t(23) =  -1.63, p = 0.09]  Hedges' g(z) = [-0.79, 0.35] |
| Broth vs. Pine | **Not equivalent** [t(23) = 1.14,  p = 0.12] and **not different** [t(23) = -1.79, p = 0.06]  Hedge’ g(z) = [-0.72, 0.04] |  | **Not equivalent** [t(23) = 1.36, p = 0.07] and **not different** [t(23) =  -1.57, p = 0.14]  Hedges' g(z) = [-0.72, 0.42] |
| Cake vs. Perfume | **Equivalent** [t(23) = 2.32, p = 0.03] and **not different** [t(23) = -0.60, p = 0.51]  Hedges' g(z) = [-0.36, 0.03] |  | **Equivalent** [t(23) = 1.98, p = 0.02]  and **not different** [t(23) = -0.94, p = 0.38]  Hedges' g(z) = [-0.64, 0.44] |
| Cake vs. Pine | **Equivalent** [t(23) = 2.42, p = 0.02] and **not different** [t(23) = -0.51, p = 0.58]  Hedges' g(z) = [-0.47, 0.22] |  | **Equivalent** [t(23) = 1.93, p = 0.02]  and **not different** [t(23) = -0.99, p = 0.30]  Hedges' g(z) = [-0.57, 0.24] |
| Perfume vs. Pine | **Equivalent** [t(23) = 2.874, p = 0.007] and **not different** [t(23) =  -0.051, p = 0.924]  Hedges' g(z) = [-0.269, 0.242] |  | **Equivalent** [t(23) = -2.81, p = 0.01]  and **not different** [t(23) = 0.11, p = 0.91]  Hedges' g(z) = [-0.54, 0.54] |

**Appendix F**

*Random effects*

Subject-specific random effects from the response time model, including intercepts and block-level deviations (slope). The widespread intercepts reflect substantial between-subject variability in baseline RT. Block-specific deviations further suggest that the effect of block was not uniform across participants. These individual differences contribute to the relatively high conditional R² (0.30) compared to the marginal R² (0.07), indicating that most of the explained variance is due to subject-level factors.

**Figure S2**

Subject-specific random effects from the response model, including intercepts and block-level and concentration-level deviations (slopes). The widespread intercepts reflect substantial between-subject variability in baseline RT. Block-specific deviations further suggest that the effect of block was not uniform across participants. While the effect of target odour concentration showed some differences between participants, this variance is likely small.

**Figure S3**

**Appendix G**

*Psychometric parameters*

| **Table XXX** | | | | | |  |
| --- | --- | --- | --- | --- | --- | --- |
| *Wilcoxon signed-rank test of PSE and JND* | | | | | |  |
|  |  | Broth | Cake | Perfume | Pine | |
| 2 | *PSE* | W = 50  p = 0.073  p_adj_ = 0.437 |  |  |  | |
|  | *JND* | W = 79  p = 0.541  p_adj_ = 0.79 |  |  |  | |
| 3 | *PSE* | W = 80  p = 0.568  p_adj_ = 1 | W = 113  p = 0.49  p_adj_ = 1 |  |  | |
|  | *JND* | W = 190  p = 0.000004***  p_adj_ = 0.00002*** | W = 165  p = 0.003**  p_adj_ = 0.01* |  |  | |
| 4 | *PSE* | W = 71  p = 0.352  p_adj_ = 1 | W = 122  p = 0.293  p_adj_ = 1 | W = 81  p = 0.595  p_adj_ = 1 |  | |
|  | *JND* | W = 190  p = 0.000004***  p_adj_ = 0.00002*** | W = 183  p = 0.00007***  p_adj_ = 0.0004*** | W = 117  p = 0.395  p_adj_ = 0.79 |  | |
| *Note: all tests done on N = 19*  * p < 0.05; ** p < 0.01; *** p < 0.001 | | | | | |  |

**Appendix H**

*Respiration*

| **Table 6** | | | |
| --- | --- | --- | --- |
| *Pearson Correlations between Inhalations and Behavioural Variables* | | | |
|  | r | 95% CI | p |
| 1. Response Behaviour^a^ | -0.055** | [-0.093, -0.017] | 0.004 |
| 2. RT | 0.821*** | [0.808, 0.833] | < 0.001 |
| 3. Pleasantness (Pre-Task) | 0.022 | [-0.204, 0.247] | 0.800 |
| 4. Pleasantness (Post-Task) | 0.097 | [-0.132, 0.315] | 0.400 |
| 5. Intensity (Pre-Task) | -0.213 | [-0.418, 0.013] | 0.060 |
| 6. Intensity (Post-Task) | -0.215 | [-0.420, 0.011] | 0.060 |
| ^a^ Point-Biserial correlation  * p < 0.05; ** p < 0.01; *** p < 0.001 | | | |
